# Editorial: Special Issue on Energy Sustainability in Marine Robotics

**DOI:** 10.3389/frobt.2022.948374

**Published:** 2022-08-10

**Authors:** Huihuan Qian, Tin Lun Lam, Fumin Zhang, Francesco Maurelli

**Affiliations:** ^1^ School of Science and Engineering, The Chinese University of Hong Kong, Shenzhen, China; ^2^ Shenzhen Institute of Artificial Intelligence and Robotics for Society, The Chinese University of Hong Kong, Shenzhen, China; ^3^ School of Electrical and Computer Engineering, Georgia Institute of Technology, Atlanta, GA, United States; ^4^ School of Computer Science and Engineering, Jacobs University Bremen, Bremen, Germany

**Keywords:** marine robotics, energy sustainability, autonomous sailboat, wave glider, autonomous underwater vehicle, marine observation

Accounting for over 70% of the Earth’s surface, the oceans have provided enriched resources and abundant space for humankind. Maritime exploration and economic development have placed tremendous challenges for contemporary technologies. Robotic systems can significantly reduce risks, increase efficiency, and extend the range of explorations in harsh marine environments. For long-term operations in the oceans, energy sustainability is a universal research theme in marine robotics, serving as foundations for locomotion, sensing, communication, control, and other related topics.

This Research Topic aims to showcase a variety of perspectives in energy sustainability so as to inspire and promote cutting edge innovations for marine robots with long-term operation capabilities. It presents a comprehensive review and original research articles in topics including efficient utilization of energies, re-charging, path planning, and state estimation. Both marine surface and underwater robots are investigated. Specifically, the following papers are included:

Sun et al. provide a comprehensive review from the energy perspectives on sailing robots for long-term operation goals. Prototypes and products developed in academia, industry, competition, and open community are categorized from aspects of sail types, hull types, electricity harvesting components, etc. Continuous progress from main contributing teams are elaborated for tracking of their R&D works. Actuations, energy harvesting, and energy management are analyzed. Various insights, e.g., motorized propellers, wave-based propulsion, balanced rudders, solar panel angles, multimode energy/E-saving management strategies, and so forth, can be used in sailboat design, control, and energy management.

Negreiros et al. present two robotic sailboats, named N-Boat and F-Boat, aiming for sustainable solutions to monitor the ocean. Energy consumption estimation and management is studied. A Restricted Boltzmann Machine is utilized to predict the sailboat’s energy consumption in a 24-h range, so as to find the most efficient way for solar panel energy harvesting. The forecasted trend of power consumption with and without solar panel fits the real data well.

Yang et al. present an unmanned sailboat platform with generic and flexible features, aiming towards the World Robotic Sailing Championship. The system is based on a 1-m class RC sailboat, with added low-cost open-source hardware module, e.g., Pixhawk, Arduino, GPS, wind direction sensor, wireless 433 MHz telegram, etc. Line-of-sight guidance strategy and PID control enables the robot to sail at way-points defined by users. Missions of fleet racing, station keeping, and area scanning validates the system and algorithms.

Gao et al. propose a novel design on the oscillating foil to improve the wave-energy-to-propulsion conversion efficiency. The main concept is that it adopts an asymmetric foil structure design and asymmetric upper and lower oscillating limit angle, which breaking through the traditional idea that the design should be symmetric for wave energy harvesting. This paper includes both the analytical and simulation analysis to evaluate the improvement of the propulsion. The proposed design has been realized and applied on a simplified wave glider, and several open sea experiments have shown the effectiveness of the proposed design.

Page et al. present an integrated navigation algorithm for reliable underwater docking and recharging of propeller-driven autonomous underwater vehicles (AUVs), so as to enhance their energy sustainability. It dynamically re-plans efficient Dubins paths from the current position towards a terminal homing location. Then the system controls an AUV by integral line of sight to follow the path. The AUV tracks the light from the hand-off location into the dock. Experiments based on two AUVs have validated the approach in lakes and pools. This approach is promising for persistent undersea operations.

Alam et al. explore the feedback planning approach, which is a function over belief space to produce an action to reach the goal belief state, aiming for energy-awareness in long-range AUVs. It attempts to overcome challenges of uncertainties on motion and sensors in the underwater environment. With a water flow pattern generated from Regional Oceanic Modeling System (ROMS) known *a-priori*, passive actions of drift are performed to save energy, while paths towards goal states are generated in different water layers through a variety of simulations.

Ismail et al. present a new form of data assimilation with a smooth variable structure filter with an application for estimating the missing states of rivers. The principal advantages of this work are 1) overcoming the missing observation and 2) improving the estimation performance for Lagrangian marine drifters. The measurements from the drifters are used in the estimation of the flow, stage, and cross section that are later used to evaluate the velocity of the sixth Lagrangian marine sensor. The convergence analysis is discussed. A comparison between the proposed method with the extended Kalman Filter shows the positive effect of the proposal.

The Guest Editors would like to thank all the authors and reviewers for their work and devotion to the Research Topic, and hope that it can inspire further research in energy sustainability for marine robotics.

